# DNAAF1 links heart laterality with the AAA+ ATPase RUVBL1 and ciliary intraflagellar transport

**DOI:** 10.1093/hmg/ddx422

**Published:** 2017-12-07

**Authors:** Verity L Hartill, Glenn van de Hoek, Mitali P Patel, Rosie Little, Christopher M Watson, Ian R Berry, Amelia Shoemark, Dina Abdelmottaleb, Emma Parkes, Chiara Bacchelli, Katarzyna Szymanska, Nine V Knoers, Peter J Scambler, Marius Ueffing, Karsten Boldt, Robert Yates, Paul J Winyard, Beryl Adler, Eduardo Moya, Louise Hattingh, Anil Shenoy, Claire Hogg, Eamonn Sheridan, Ronald Roepman, Dominic Norris, Hannah M Mitchison, Rachel H Giles, Colin A Johnson

**Affiliations:** 1 Leeds Institute of Biomedical and Clinical Sciences, Faculty of Medicine & Health, University of Leeds, Leeds LS9 7TF, UK; 2 Department of Nephrology and Hypertension; 3 Department of Medical Genetics, University Medical Center, Utrecht, 3508 GA, The Netherlands; 4 Genetics and Genomic Medicine Programme, UCL Great Ormond Street Institute of Child Health, University College London, London WC1N 1EH, UK; 5 NIHR Great Ormond Street Hospital Biomedical Research Centre, London WC1N 1EH, UK; 6 Mammalian Genetics Unit, MRC Harwell Institute, Harwell Campus, Oxfordshire OX11 0RD, UK; 7 Leeds Genetics Laboratory, Leeds Teaching Hospitals NHS Trust, Leeds LS9 7TF, UK; 8 PCD Diagnostic Team and Department of Paediatric Respiratory Medicine, Royal Brompton and Harefield NHS Trust, London SW3 6NP, UK; 9 School of Medicine, University of Dundee, Dundee DD1 9SY, UK; 10 Department of Zoology, Faculty of Science, Benha University, Benha, Egypt; 11 Manchester Royal Infirmary, Manchester M13 9WL, UK; 12 Developmental Biology and Cancer Programme, UCL Great Ormond Street Institute of Child Health, University College London, London WC1N 1EH, UK; 13 Department for Ophthalmology, Institute for Ophthalmic Research and Medical Bioanalytics Core, University of Tübingen, 72074 Tübingen, Germany; 14 Paediatric Cardiology Department, Great Ormond Street Hospital for Children NHS Foundation Trust, London WC1N 3JH, UK; 15 Department of Paediatrics, Luton and Dunstable Hospital NHS Trust, Luton LU4 0DZ, UK; 16 Department of Paediatrics, Bradford Teaching Hospitals NHS Trust, Bradford BD9 6RJ, UK; 17 Department of Human Genetics, Radboud University Medical Center, 6500HB Nijmegen, The Netherlands

## Abstract

DNAAF1 (LRRC50) is a cytoplasmic protein required for dynein heavy chain assembly and cilia motility, and *DNAAF1* mutations cause primary ciliary dyskinesia (PCD; MIM 613193). We describe four families with *DNAAF1* mutations and complex congenital heart disease (CHD). In three families, all affected individuals have typical PCD phenotypes. However, an additional family demonstrates isolated CHD (heterotaxy) in two affected siblings, but no clinical evidence of PCD. We identified a homozygous *DNAAF1* missense mutation, p.Leu191Phe, as causative for heterotaxy in this family. Genetic complementation in *dnaaf1*-null zebrafish embryos demonstrated the rescue of normal heart looping with wild-type human *DNAAF1*, but not the p.Leu191Phe variant, supporting the conserved pathogenicity of this *DNAAF1* missense mutation. This observation points to a phenotypic continuum between CHD and PCD, providing new insights into the pathogenesis of isolated CHD. In further investigations of the function of DNAAF1 in dynein arm assembly, we identified interactions with members of a putative dynein arm assembly complex. These include the ciliary intraflagellar transport protein IFT88 and the AAA+ (ATPases Associated with various cellular Activities) family proteins RUVBL1 (Pontin) and RUVBL2 (Reptin). Co-localization studies support these findings, with the loss of RUVBL1 perturbing the co-localization of DNAAF1 with IFT88. We show that RUVBL1 orthologues have an asymmetric left-sided distribution at both the mouse embryonic node and the Kupffer’s vesicle in zebrafish embryos, with the latter asymmetry dependent on DNAAF1. These results suggest that DNAAF1-RUVBL1 biochemical and genetic interactions have a novel functional role in symmetry breaking and cardiac development.

## Introduction

Congenital heart disease (CHD) is the most common congenital defect affecting 0.5–0.8% of all live births (1). Complex forms of CHD carry a high morbidity and mortality and are associated with disorders of laterality. *Situs solitus* is the term used to describe a normal arrangement of body anatomy, whereas *situs inversus* is the mirror image of this arrangement. A failure of asymmetry of the unpaired organs is termed *situs ambiguus* or heterotaxy, and is associated with major CHD. Heterotaxy is rare, occurring in 1: 10,000 pregnancies (2), and the genetic aetiology is complex. Mutations in more than 15 genes have been described as causes of inherited forms of heterotaxy, but the majority of cases (80%) are thought to be sporadic (3). Isomerism refers to a defect in the anatomy of paired organs that usually have distinct left and right forms, but in isomerism are placed in a mirror image formation (4). Heterotaxy and laterality defects are frequent clinical features of ciliopathies, disorders in the structure or function of cilia, especially those affecting the motile cilia (5,6).

Cilia are small microtubule-based organelles that play sensory roles responding to chemical, morphogenic and mechanical stimuli, as well as roles in cell and fluid motility. The majority of vertebrate cell types express a single immotile sensory primary cilium characterised by an axonemal core of nine microtubule doublets in a 9 + 0 arrangement. However, multiple specialized motile cilia are present on differentiated epithelial cell-types of the brain, respiratory tract and Fallopian tubes. Axonemal cores of motile cilia consist of nine microtubular doublets surrounding a central pair of microtubules in a 9 + 2 arrangement. In motile cilia, inner and outer dynein motor ‘arms’ (IDA and ODA) are attached to the peripheral microtubules that act as molecular motors to provide the force required for ciliary motility (7,8). Radial spokes connect the outer microtubules to the central pair to provide a signal transduction scaffold that regulates peripheral motors from the centre (9,10). A further type of specialized motile cilia are present at the embryonic node, with a 9 + 0 microtubule structure. These nodal cilia are involved in the creation of a leftward fluid flow at the embryonic node, a key step in breaking left-right symmetry in the early embryo (11).

In motile cilia, dynein arms are incorporated into the axoneme as pre-assembled building blocks, which requires preassembly of the IDAs and ODAs in the cytoplasm and their subsequent transport into the ciliary axoneme, although the mechanism by which this occurs is not known. ODAs are responsible for maintaining ciliary beat frequency and IDAs are required for normal waveform. There are species-specific differences in dynein arm composition, but human respiratory cilia ODAs consist of a globular head domain (heavy chains HC beta [DNAH11/DNAH9] and HC gamma [DNAH5]), intermediate domain (DNAI1, DNAI2) and a docking complex (CCDC114, CCDC151, ARMC4, TTC25), involved in anchoring the complex to the neighbouring microtubule doublet (8,12,13). Associated with this complex are multiple light chains required for assembly, stability and motility (7).

Primary ciliary dyskinesia (PCD; MIM 244400) is predominantly an autosomal recessive disorder of motile cilia with an incidence of 1: 15,000. Impaired mucociliary clearance leads to a disorder of the sinopulmonary tract, presenting as neonatal respiratory distress, chronic respiratory tract infections, bronchiectasis and sinusitis. The link between disorders of laterality and PCD has been long-established. *Situs inversus* is present in around 50% of individuals affected with PCD (termed Kartagener syndrome), due to the role of nodal cilia in the creation of asymmetry at the embryonic node (11) CHD is also common in PCD, occurring in 3.5–6% of affected individuals (14). The association between CHD and PCD is further supported by the demonstration of ciliary dysfunction in a cohort of patients with heterotaxy and complex CHD (15,16). Mutations in genes encoding dynein assembly factors, radial spoke components, components of the ODA and dynein regulatory complex have all been shown to cause PCD.

*DNAAF1* (also known as *LRRC50*) is a human PCD disease gene (17,18) and affected individuals have combined ODA and IDA defects (17). *DNAAF1* is orthologous to *ODA7* in *Chlamydomonas* and was initially identified as a dynein assembly factor, with mutations in *ODA7* causing reduced ciliary beat frequency and a block in outer dynein arm assembly (19,20). Two different zebrafish *dnaaf1*^*−*^^*/*^^*−*^ mutants develop typical motile cilia defects that include pronephric cysts, and have randomized heart jogging and abnormal polarity of the visceral organs in more than 50% of mutants (21,22). The *dnaaf1* mutants have ultrastructural abnormalities of the dynein arms (lacking either ODA, or both IDA and ODA) and have misalignment of the outer microtubules, indicating that the microtubular architecture in these mutants is unstable (21,23). In human respiratory cells with mutations in *DNAAF1*, specific dynein heavy, intermediate and light chains (DNAH5, DNAH9, DNAI2 and DNALI1) were absent from the ciliary axoneme (17), supporting the function of DNAAF1 as a dynein assembly factor. However, the possible mechanisms by which DNAAF1 mediates ODA pre-assembly and targeting of dyneins to the axoneme is largely unknown.

In this study, we present a series of families affected by mutations in *DNAAF1*. Three families have typical PCD phenotypes with a high co-morbidity of cardiac defects, but a fourth family has isolated CHD (heterotaxy) in two affected siblings with no clinical evidence of PCD. We interpret a homozygous *DNAAF1* missense mutation as causative for heterotaxy in the fourth family including by genetic complementation assays in a *dnaaf1* null zebrafish model. We identify and confirm biochemical and functional interactions of DNAAF1 with IFT88 and RUVBL1 (Pontin), representing novel members of a putative dynein assembly complex. We also show that RUVBL1 has an early left-handed asymmetric distribution at the mouse embryonic node, and that this asymmetry at the Kupffer’s vesicle in zebrafish embryos is dependent on DNAAF1. These results expand the functional roles of RUVBL1 to include symmetry-breaking and cardiac development.

## Results

### Clinical features and genotype-phenotype correlations

**Figure 1.**
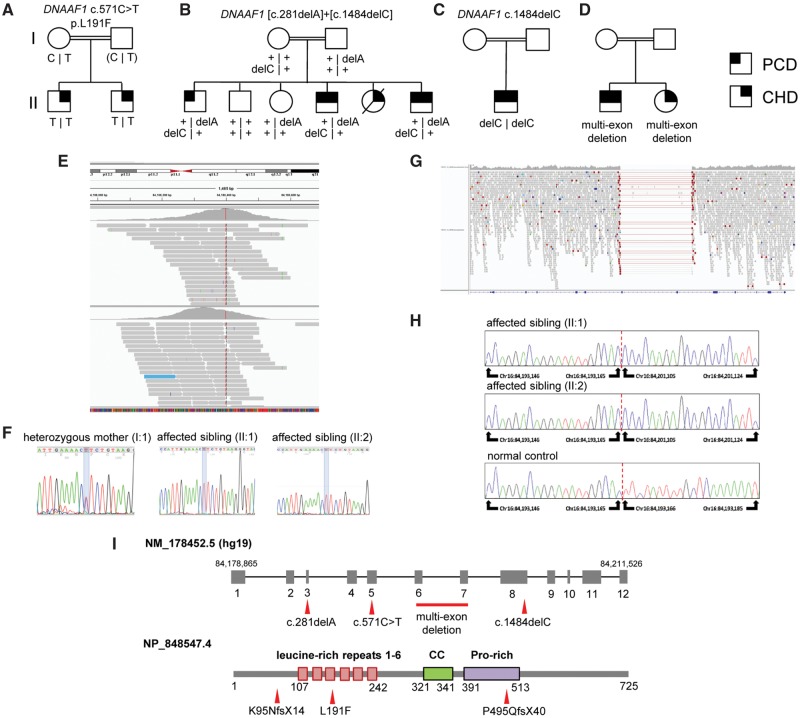
Segregation and location of *DNAAF1* mutations in PCD and CHD families: (**A–D**) Pedigrees of families A–D where shading in the upper left quadrant indicates a diagnosis of PCD and shading in the upper right quadrant indicates a diagnosis of CHD. (**E**) Integrative Genomics Viewer (IGV) readout with aligned whole-exome sequence reads shown in grey, indicating (in red) the *DNAAF1* variant c.571C > T, which is homozygous in both affected individuals of Family A. (**F**) Electropherograms of individuals in Family A showing (i) the mother of affected individuals II: 1 and II: 2, showing the variant in heterozygous form, (ii) affected male II: 1 and (iii) affected male II: 2, showing the variant in homozygous form. The *DNAAF1* variant is marked with blue shading. (**G**) IGV readout of whole-genome sequencing reads in Family D, showing a homozygous deletion of 7.9 kilobases in *DNAAF1*, corresponding to an absence of aligned reads spanning chr16: 84, 193, 165–84, 201, 105. (**H**) Genomic Sanger sequencing assay to show the breakpoints of a homozygous multi-exon deletion of 7940 bases in *DNAAF1* (chr16[GRCh37]: g84193166_8201104del) for Family D. (**I**) Position of mutations (red arrowheads) in the *DNAAF1* gene (top) and protein (bottom), showing newly described mutations. The c.571C > T p.Leu191Phe mutation associated with isolated heterotaxy occurs in the leucine-rich repeat (LRR) protein domain. “CC” indicates a single coiled-coil domain and “Pro-rich” indicates a region of compositional bias.

Whole exome sequencing (WES) was performed in three separate centres as part of studies to determine the genetic basis of CHD and PCD (Leeds Institute of Biomedical and Clinical Sciences, UCL Great Ormond Street Institute of Child Health and the Leeds Genetics Laboratory). This identified putative disease-causing mutations in *DNAAF1* in four families with complex congenital heart disease, with or without a clinical diagnosis of PCD (Fig. 1A–D). All four families are from consanguineous Pakistani ethnic backgrounds, living in the UK.

Family A has two children affected with complex CHD (Fig. 1A). The affected male child II: 1 has complete atrioventricular septal defect (AVSD) with significant AV valve regurgitation, double outlet right ventricle (DORV), pulmonary atresia, right atrial isomerism and right pulmonary artery stenosis. An initial Blalock-Taussig shunt and a subsequent bidirectional cavopulmonary shunt has been performed. His affected sibling II: 2 has heterotaxy, described as dextrocardia, right atrial isomerism with a left-sided liver, right-sided stomach and right-sided polysplenia. There is DORV, with the pulmonary artery to the right and the aorta to the left. The aortic arch is rightward, and there is a common atrium. Both ventricles are small and there is a large inlet ventricular septal defect (VSD) and severe subpulmonary stenosis. This child has had a Glenn shunt performed. Neither of the affected individuals suffered from neonatal respiratory distress, recurrent chest infections, chronic cough or chronic nasal symptoms. II: 1 has had recurrent episodes of otitis media. Their reduced exercise tolerance has been ascribed to CHD. They are non-dysmorphic and have normal learning.

Family B has three of six children confirmed as affected with PCD, with a fourth child (II: 5) who died aged 2 months of cardiac complications before a presumptive diagnosis of PCD could be confirmed (Fig. 1B). Diagnosis of PCD in the surviving children was made in the clinical setting following low nitric oxide (NO) measurements and typical respiratory features of PCD along with the observation of completely static nasal cilia observed by high speed video imaging (0Hz ciliary beat frequency). Individual II: 1 had an NO level of 40 ppb (10 nl/min). Diagnostic transmission electron microscopy (TEM) analysis of the cilia was abnormal in the three siblings II: 1, II: 4 and II: 6, showing the absence of ciliary outer dynein arms in 84%, 92% and 100% of cilia cross-sections respectively accompanied by occasionally absent inner dynein arms (affecting 21, 22 and 30% of cross-sections, respectively). The oldest child II: 1 has PCD with normal *situs* whilst the other two affected surviving children (II: 4, II: 6) both have *situs inversus* with asplenia and a cardiac defect of right atrial isomerism (RAI). In addition to RAI, II: 4 has the most severe phenotype and is on home oxygen affected with AVSD, pulmonary stenosis and anomalous pulmonary venous drainage. Cardiac surgery was performed in early infancy consisting of a Blalock-Taussig shunt aged 3 days, a bilateral-directional Glenn shunt procedure at 2 years old and completion of total cavopulmonary connection aged 6. In addition to RAI, II: 6 also has dextrocardia (azygous vein to left sided superior vena cava) and anomalous pulmonary venous drainage, but no intervention was yet required. Deceased child II: 5 was affected with RAI and the same multiple cardiac defects as II: 4, but died aged 2 months of cardiac complications before a presumptive diagnosis of PCD could be confirmed.

Family C consists of a single male affected with clinically confirmed PCD (Fig. 1C). He has dextrocardia, longstanding otitis media and asthenospermia.

In Family D, two siblings are affected (Fig. 1D). The first (II: 1) had suffered from neonatal respiratory distress and persistent rhinorrhea from birth. He also had *situs inversus*, dextrocardia, congenitally-corrected transposition of the great vessels, perimembranous ventricular septal defect (VSD) and tricuspid regurgitation. Nasal ciliary brushing confirmed a diagnosis of PCD with static cilia, and a lack of ODA and IDA. The second (II: 2) had a history of chronic cough, static cilia as observed by high-speed videomicroscopy, and absent ODA and IDA identified by TEM. This affected individual had no history of cardiac disease.

### Whole exome sequencing identifies *DNAAF1* mutation as a cause of isolated heterotaxy

Whole exome sequencing (WES) was performed in all four families. Family A was screened at the Leeds Institute of Biomedical and Clinical Sciences, and Families B-D were screened as cases referred to the diagnostic PCD services based at UCL Great Ormond Street Institute of Child Health (Family B) and the Leeds Genetics Laboratory (Family C and D). Similar variant calling pipelines were followed in all analysis, as previously published (24,25) and as described below for Family A.

Following alignment to the reference sequence (GRCh37) and variant calling, variants were retained that were rare (<1% MAF in ExAC, dbSNP, EVS and 3000 ethnically-matched control exomes), functional (following removal of synonymous, non-coding, intronic and intergenic variants) and consistent with inheritance pattern in the family (bialleic in affected individuals, but not biallelic in parents or unaffected siblings). In Family A, a SNP array identified a 1Mb region of homozygosity on chromosome 16. Only two variants remained following filtering in Family A (Supplementary Material, Table S1) and the variant with the highest pathogenicity score, c.571C > T in *DNAAF1* (NM_178452), also occurred within the homozygous region. This variant predicts the missense mutation p.Leu191Phe in DNAAF1. Pathogenic variants were not identified in any other known PCD genes. CNV analysis was performed on exome data in Family A using the ExomeDepth program, but no pathogenic CNVs were identified following removal of common variation. The c.571C > T mutation was confirmed by Sanger sequencing and was homozygous in both patients and heterozygous in their mother (paternal DNA was not available for testing; Fig. 1E and F). The c.571C > T (p.Leu191Phe) missense mutation was extremely rare amongst healthy individuals. It was not present in the ExAC or gnomAD databases and only two heterozygotes were present in 3000 ethnically-matched control samples. A further 19 patients affected with heterotaxy were investigated for mutations in *DNAAF1*, but no further pathogenic mutations were identified. The p.Leu191Phe mutation in Family A falls within a region of leucine-rich repeat (LRR) domains in DNAAF1 and affects a highly conserved leucine residue (Fig. 1I).

In Family B, heterozygous frameshift mutations in *DNAAF1* were identified: c.281delA predicted to cause the frameshift change p.Lys95Asnfs*14, and c.1484delC predicted to cause the frameshift change p.Pro495Glnfs*40 (Fig. 1B). These mutations segregated with disease in all the available family members and were confirmed by Sanger sequencing. Both variants were absent the ExAC or gnomAD databases. The affected individual in Family C shared the same *DNAAF1* mutation c.1484delC (p.Pro495Glnfs*40) in the homozygous state, which was also confirmed by Sanger sequencing (Fig. 1C). WES data suggest that this mutation appears to be carried on a common disease haplotype for Family B and C which both share a Pakistani ethnic origin.

Both affected individuals in Family D shared a homozygous multi-exon deletion of 7940 bases in *DNAAF1* (chr16[GRCh37]: g84193166_8201104del). This deletion was identified from exome data following the observation that no sequence reads had mapped to the targeted exons (Fig. 1G). Breakpoints were subsequently identified by whole genome sequencing (WGS), and use of a breakpoint-spanning Sanger sequencing assay confirmed the presence and zygosity of the deletion in both affected siblings (Fig. 1H).

### Investigation of PCD in Family A

Investigations were performed to identify or refute a PCD phenotype in Family A. NO measurements in individual II: 1 showed a level of 254 ppb (63 nl/min) in the right nostril (normal range) and 121 ppb (30 nl/min) in left nostril (low, but nostril blocked by upper respiratory tract infection; ambient level of 17 ppb). Ciliary beat frequency for individual II: I was 8.7Hz (SD 2.2Hz), within the normal range, compared to 9.1Hz (SD 2.1Hz) in a control sample taken concurrently. Video microscopy of nasal epithelial cells in individual II: 1 identified a sparsely ciliated epithelium. Cilia effectively cleared surrounding debris, with some thick mucus impeding the cilia beat in places. There was a mixed beat pattern with some slow, dyskinetic and static patches. This sample was collected concurrently as a sample from a healthy control, and video microscopy was performed after the transport of the samples at room temperature for several hours. The control sample had long, well-ciliated strips of epithelium and the cilia were seen effectively clearing the surrounding debris. The ciliary beat was mostly coordinated, but not recorded as entirely normal because it was also mixed in places with some slow, dyskinetic and static patches (see Supplementary Movies). In affected individual II: 1, only 10 ciliary cross-sections could be assessed by TEM, but these all showed normal ODA and IDA to be present. Overall, there was no evidence to support a diagnosis of PCD in individual II: 1 from Family A on clinical testing. Parental consent for ciliary brushings was not given for individual II: 2 in Family A.

### Genetic complementation of heart looping defects in *dnaaf1*^*−/−*^ zebrafish embryos by wild-type but not p.Leu191Phe mutant DNAAF1

**Figure 2.**
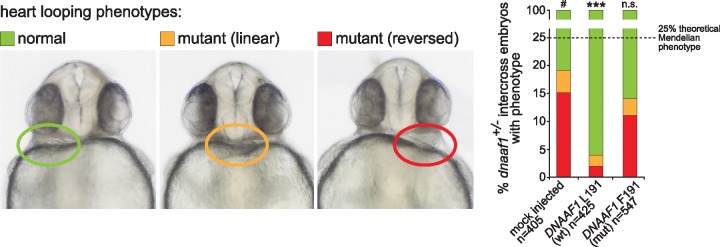
Genetic complementation of heart looping defects in *dnaaf1^−/−^* mutant zebrafish embryos: Zebrafish embryos from *dnaaf1^+/−^* heterozygote-heterozygote inter-crosses were either mock injected (control, *n =* 405 embryos), or injected with mRNA expressed from human wild-type (wt, *n =* 425 embryos) and p.Leu191Phe-mutant (mut, *n =* 547 embryos) pCS2+ *DNAAF1* constructs. In all cases, a mixture of normal looping (green), reversed looping (red) or ‘linear’ looping indicating a lack of heart looping (orange) were seen and representative examples are shown at 72 h post fertilization (left panels). On the right, the bar graph quantifies the three positions of heart looping in all three experiments, showing that phenotypic rescue is seen after injection of wild-type but not mutant RNA. n.s.= not significant; *** *P *<* *0.001 (one-way ANOVA, *n =* 3 biological replicates). The dashed line indicates the 25% expected Mendelian ratio of embryos that should be homozygous mutant and manifest a phenotype.

*dnaaf1* ^*−/−*^ (*dnaaf1^hu255h^*) mutant zebrafish, with a T > A point mutation creating a premature stop codon p.Leu88* in the zebrafish orthologous gene, were previously described (21). Mutant embryos have pronounced ventral body curvature, pronephric cysts and pronephric duct dilatation which are all typical features of PCD zebrafish models. Also characteristic for PCD, is the randomisation of heart looping in mutants caused by static cilia in the Kupffer’s vesicle. Homozygous mutants die at 8dpf with severe oedema. To interpret the possible pathogenicity of the c.571C > T (p.Leu191Phe) *DNAAF1* mutation as causative for isolated CHD phenotype in Family A, we performed genetic complementation assays in the *dnaaf1* mutant zebrafish line. mRNA of mutant (c.571T) and wild-type (c.571C) human *DNAAF1* was injected into zebrafish clutches from *dnaaf1^+/−^* intercrosses. In mock-injected negative control clutches, about 16% of embryos showed reversed heart looping and central heart phenotypes (Fig. 2). This is fewer than the expected Mendelian ratio of 25% for the homozygous mutant phenotype, because in *dnaaf1-*deficient embryos the laterality of Kupffer’s vesicle is randomized (21). Significant rescue of left-right axis defects (reversed or no heart looping) was demonstrated at 72 h post fertilization in *dnaaf1^+/−^* inter-crossed embryos injected with human wild-type *DNAAF1* transcript. However, rescue was not significant for embryos injected with the mutant mRNA, supporting the pathogenicity of the c.571C > T mutation (Fig. 2). By comparison with wild-type zebrafish lines kept in the same conditions, the background level of *situs* defects is <5% (Jeroen Bakkers, personal communication).

### DNAAF1 interacts with IFT88, and the AAA+ ATPases RUVBL1 and RUVBL2

**Figure 3.**
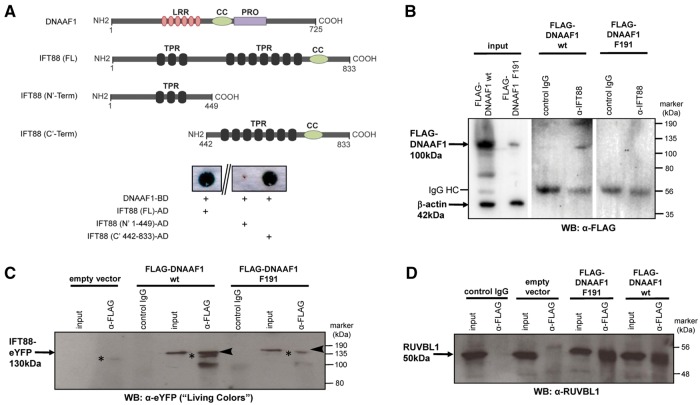
Interactions of DNAAF1 with IFT88 and RUVBL1: (**A**) Schematic diagram to show DNAAF1 and IFT88 domains. Full-length DNAAF1 contains 6 leucine-rich repeats (LRR), a coiled-coil domain (CC) and a proline-rich domain (PRO). IFT88 contains 3 N-terminal and 7 C-terminal tetratricopeptide repeats (TPR). A dedicated yeast two-hybrid for DNAAF1-BD demonstrates interaction with full-length IFT88 and N-terminally truncated IFT88 (442–833). The interaction is not observed in C-terminal truncated IFT88 (1–449). (**B**) Confirmation of the interaction between DNAAF1 and IFT88, demonstrated by co-immunoprecipitation assays comprising over-expression of wild-type (wt; p.Leu191) or mutant (p.Phe191) FLAG-DNAAF1, pull-down of endogenous IFT88 by anti-IFT88 antibody and western blot (WB) with anti-FLAG. Input whole cell extracts indicate significantly reduced levels of DNAAF1 mutant (p.Phe191) compared to wild-type protein, with β-actin used a loading control. Negative control IgG lanes and IgG heavy chain (HC) bands are indicated. (**C**) Co-immunoprecipitation assays of over-expressed wild-type (wt; p.Leu191) or mutant (p.Phe191) FLAG-DNAAF1 and IFT88-eYFP, pull-down with anti-FLAG antibody and western blot (WB) with anti-GFP “Living Colors” antibody. Input whole cell extract and irrelevant control IgG lanes are indicated, with empty vector as the negative control. The largest IFT88 band that is immunoprecipitated by the wild-type DNAAF1 protein, is indicated by the arrowheads, and this interaction is lost with the mutant DNAAF1 protein. Non-specific protein bands, indicated by the asterisks, are likely to be due to cross-reaction of the secondary antibody with the anti-FLAG IgG used in these coIPs. (**D**) Interaction between DNAAF1 and RUVBL1 (Pontin) shown after over-expression of wildtype (p.Leu191) or mutant (p.Phe191) FLAG-DNAAF1, pull-down with anti-FLAG antibody and western blot (WB) blot with anti-RUVBL1 antibody.

To further model the pathogenic effect of the c.571C > T (p.Leu191Phe) missense mutation and to understand the potential functions of DNAAF1, we began by performing screens for interactors of DNAAF1. A GAL4 yeast two-hybrid screen, using an oligo dT-primed human retinal cDNA library, was performed to identify further protein-protein interactions of wild-type DNAAF1. The single positive clone identified by this screen encoded intraflagellar transport protein IFT88. Confirmation was performed with a dedicated “one-to-one” yeast two-hybrid assay (Fig. 3A), showing that DNAAF1 interacted with both full length and N’-terminally truncated IFT88, but not C’-terminally truncated IFT88. Co-immunoprecipitation (coIP) assays confirmed that both endogenous IFT88 (Fig. 3B) and exogenously-expressed IFT88 (Fig. 3C) interacted with wild-type DNAAF1 (p.Leu191). Interactions of IFT88 with mutant DNAAF1 (p.Phe191) were either absent or diminished (Fig. 3B and C) due to either reduced expression levels or reduced stability of the mutant protein (Fig. 3B).

We then used streptavidin-II/FLAG tandem affinity purification (SF-TAP) of N-terminal TAP-tagged wild-type and p.Phe191 mutant DNAAF1 to identify interacting proteins by mass spectrometry (Table 1, Supplementary Material, Table S2). In this assay, the peptide counts corresponding to the interacting proteins were highly reproducible between biological replicates suggesting that interactions were stable. As expected, DNAAF1 was pulled down in all assays, confirming moderate levels of protein expression, although levels were lower for the mutant p.Phe191 protein again suggesting reduced expression levels or stability. Consistent with the possible instability of the mutant protein, several heat shock proteins (HSPA1A, HSPA5, HSPA8, HSPA9, HSPA6 and HSP90AA1) were more strongly associated with the mutant p.Phe191 DNAAF1 protein than the wild-type protein (Table 1, Supplementary Material, Table S2). Both RUVBL1 and RUVBL2 (also known as Pontin and Reptin, or RVB1 and RVB2), members of AAA+ (ATPases Associated with various cellular Activities) protein family, associated with the wild-type DNAAF1 protein, and the RUVBL1 interaction was reduced by the DNAAF1 p.Phe191 mutation. CoIPs assays confirmed the interaction of both endogenous RUVBL1 (Fig. 3D) and RUVBL2 (Supplementary Material, Fig. S1) with DNAAF1.

**Table 1.** Proteins identified by SF-TAP pull-downs with DNAAF1 p.Leu191 wild-type or p.Phe191 mutant proteins: Unique peptides (>5 for each protein) were identified by mass spectrometry, in at least two out of three biological replicates. Results are ordered from the highest to lowest number of pulled-down peptides. Statistically significant differences in peptide numbers for wild-type compared to mutant pull-downs are indicated (Chi-squared test; n.s. not significant, *P =* *0.05, ****P =* *0.0001; for *n =* 3 biological replicates). Log-fold changes of mutant to wildtype protein levels are also shown, where a negative value indicates an increased association with wildtype protein and a positive value indicates an increased association with the mutant protein. A “+” indicates exclusive binding of that protein species to the p.Phe191 mutant protein. Several proteins of the chaperonin containing TCP1 (CCT) complex also associated with the mutant protein only, but have been removed for brevity. Further details are available in Supplementary Material, Table S2

Protein symbol	Protein function	UniProt accession number	Protein size (kDa)	No. unique peptides associated with wild-type protein	Log-fold change (mutant vs wild-type protein)	Chi-squared test (mutant vs wild-type protein)
DNAAF1	Dynein assembly factor	Q8NEP3	80	378	−0.058	*
HSPA9	Heat-shock protein	P38646	74	121	−0.004	n.s.
HSPA8	Heat-shock protein	P11142	71	89	+0.181	****
HSPA1A	Heat-shock protein	P08107	70	86	+0.186	****
HSPA5	Heat-shock protein	P11021	72	53	+0.075	n.s.
ACACA	Acetyl co-a carboxylase	Q13085	266	50	−0.344	n.s.
RUVBL1	Pontin	Q9Y265	50	28	−0.049	n.s.
TUBB4B	Tubulin	P68371	50	24	+0.602	****
TUBA1B	Tubulin	P68363	50	20	+0.531	****
RUVBL2	Reptin	Q9Y230	51	19	+0.083	****
ACTB	Beta actin	P60709	42	15	−0.030	n.s.
UBA52	Ubiquitin-ribosomal protein fusion product	P62987	15	10	+0.146	n.s.
PC	Pyruvate carboxylase	P11498	130	8	+0.026	n.s.
TUBB	Tubulin	P07437	50	8	+0.495	****
SLC25A5	Solute carrier	P05141	33	5	+0.643	****
EEF1A1	Eukaryotic translation elongation factor	P68104	50	4	+0.176	n.s.
CCT6A	chaperonin containing T complex polypeptide	P40227	58	3	+0.493	n.s.
ATP5A1	ATP synthase	P25705	60	3	+0.523	n.s.
HSPA6	Heat-shock protein	P17066	71	0	+	n/a
HSP90AA1	Heat-shock protein	P07900	85	0	+	n/a
SUGT1	Suppressor of G2 allele of SKP1	Q9Y2Z0	41	0	+	n/a

### DNAAF1 localization to the ciliary basal body is perturbed by loss of RUVBL1

In ciliated cells, DNAAF1 is dynamically localized throughout the cell cycle. DNAAF1 is a cytoplasmic protein that is localized to the basal body of the primary cilium and maintains centrosome association throughout the cell cycle, with transient localization to the mid-body and condensed chromosomes during metaphase (23). In ciliated hTERT-RPE1 cells, endogenous DNAAF1 localized distal to RPGRIP1L, but co-localized with gamma-tubulin and centrin-3, confirming a specific basal body localization that is adjacent to the transition zone (Supplementary Material, Fig. S2A). Over-expressed FLAG-tagged wild-type p.Leu191 and mutant p.Phe191 DNAAF1 had a more diffuse cytoplasmic distribution, but also localized to the ciliary base (Supplementary Material, Fig. S3A and B). Over-expressed IFT88 partially co-localized with both wild-type and mutant DNAAF1 at the ciliary base (Supplementary Material, Fig. S3A and B), and in agreement with the biochemical interactions between IFT88 and DNAAF1 (Fig. 3A–C). Endogenous RUVBL1 had a predominantly nuclear localization and did not appear to extensively co-localize with DNAAF1 (Supplementary Material, Fig. S2B).

**Figure 4.**
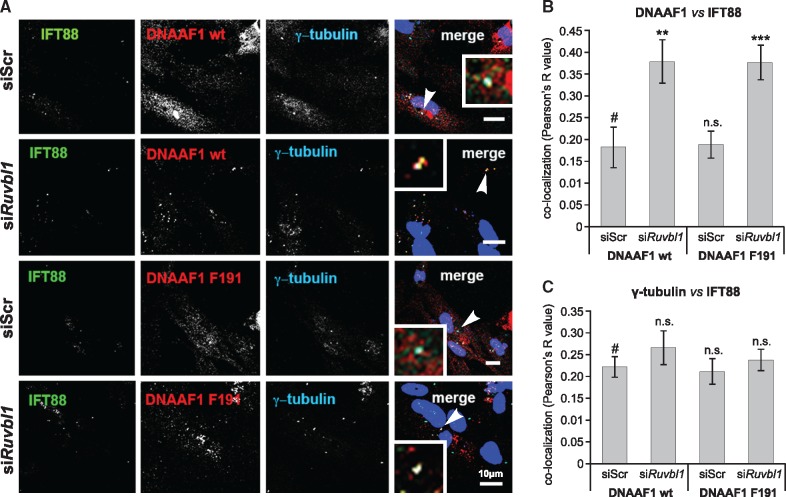
Disruption of co-localization of wild-type DNAAF1 with IFT88 after *RUVBL1* knockdown: (**A**) Immunofluorescence confocal microscopy of ciliated hTERT-RPE1 cells showing co-localization of over-expressed DNAAF1 p.L191 wild-type (wt) or p.F191 mutant proteins (red) with endogenous IFT88 (green) at cilia, and basal bodies marked by γ-tubulin (blue). Examples of co-localization, indicated by arrowheads, are shown in the magnified insets. Scale bars = 10 μm. (**B**) Knockdown of *RUVBL1* disrupts the co-localization (expressed as Pearson's R value) between IFT88 and either wild-type or mutant DNAAF1. Statistical significance of pair-wise comparison with scrambled (Scr) negative control siRNA (#) indicated by: n.s. not significant; ** *P <* 0.01; *** *P <* 0.001 (paired Student t-test; error bars indicate s.e.m. for *n =* 3 biological replicates). (**C**) Co-localization of IFT88 with γ-tubulin as for (B), showing no significant effect of *RUVBL1* knockdown.

Since the RUVBL1/RUVBL2 complex has been implicated in the assembly of both protein and ribonucleoprotein complexes (26), we reasoned that RUVBL1 and RUVBL2 could mediate the pre-assembly of a putative DNAAF1 protein complex that might include IFT88 as a component. To test this, we depleted the levels of either RUVBL1 or RUVBL2 by siRNA knock-down in hTERT-RPE1 cells. Knock-down was assessed by real-time PCR (Supplementary Material, Fig. S4A) and western blotting (Supplementary Material, Fig. S4B), showing more extensive loss of RUVBL1 at the transcript and protein levels than for RUVBL2. We therefore focused on *RUVBL1* knock-downs in hTERT-RPE1 cells, and quantified the degree of co-localization between endogenous IFT88 and DNAAF1, and IFT88 with γ-tubulin (Fig. 4A). *RUVBL1* knockdown significantly increased the co-localization of endogenous IFT88 with either wild-type DNAAF1 (p.Leu191) or mutant DNAAF1 (p.Phe191) (Fig. 4B), but had no effect on the co-localization of IFT88 with γ-tubulin (Fig. 4C). This suggests that reduced levels of RUVBL1 perturb the localization or transport of DNAAF1, without affecting overall ciliary ITF. Both wild-type and mutant DNAAF1 had similar levels of co-localization with ciliary IFT88 (Fig. 4B), suggesting that they have a similar functional association with RUVBL1.

### Asymmetric distribution of RUVBL1 at the zebrafish Kupffer’s vesicle and mouse embryonic node is dependent on DNAAF1

**Figure 5.**
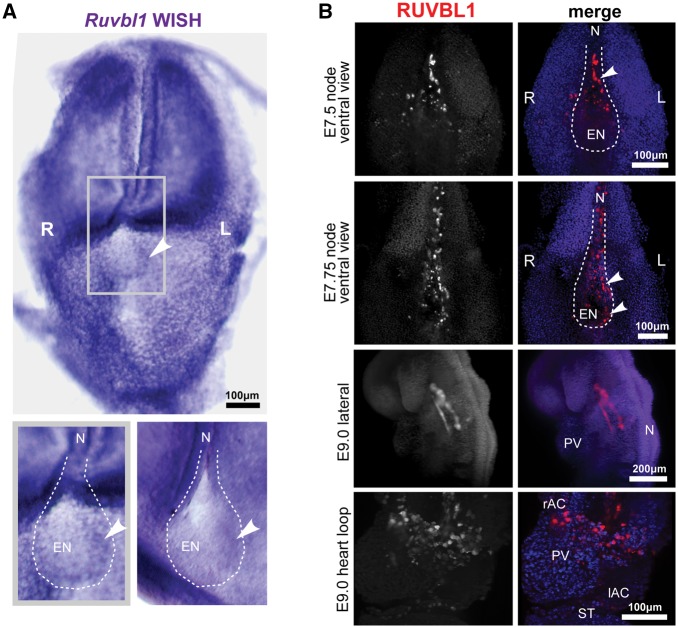
Left-sided asymmetric expression of RUVBL1 at the mouse embryonic node: (**A**) whole-mount *in situ* hybridization (WISH) of *Ruvbl1* probes for whole-mount head-fold stage wild-type C3H/HeH mouse embryos showing widespread, ubiquitous *Ruvbl1* expression and left-sided asymmetric distribution at the embryonic node. Grey frame indicates magnified inset (lower left) with a second example at lower right, with *Ruvbl1* expression indicated by arrowheads. Scale bar = 100 μm. (**B**) Light-sheet microscopy images of whole-mount immunofluorescence for RUVBL1 (red) and DAPI (blue) in the wild-type C57BL/6 mouse E7.25 and E7.5 (late head fold and 2–4 somite stage) embryonic node, and E9.0 (6–8 somite stage) vasculature and heart loop. Left (L) and right (R) sides are indicated. RUVBL1 is asymmetrically distributed at the murine embryonic node (upper two panels). RUVBL1 is present in a widespread distribution throughout the atrial chambers, primitive ventricle and developing vasculature (lower two panels). Scale bars = 100 or 200 μm, as indicated. Abbreviations: rAC and IAC, right and left atrial chambers; EN, embryonic node; L, left; N, notochord; PV, primitive ventricle; R, right; ST, septum transversum.

Since RUVBL1 could mediate an apparent functional association between DNAAF1 and IFT88, we next asked if RUVBL1 could mediate normal laterality phenotypes through DNAAF1. We first assessed the localization of *Ruvbl1* transcripts in the mouse embryonic node using whole-mount *in situ* hybridization (WISH). In head-fold stage embryos, *Ruvbl1* was ubiquitously expressed, but in the embryonic node a low but discernible asymmetric left-sided distribution was evident (Fig. 5A). We next assessed the localization of RUVBL1 protein in wild-type mouse embryos using light-sheet immunofluorescence microscopy. In late head fold and early somite stage embryos (E7.25-E7.5), high levels of RUVBL1 expression exhibited a left-sided distribution in the node, with only moderate expression in the surrounding tissues (Fig. 5B). At later stages of embryogenesis (6–8 somite pairs, E9.0), asymmetric RUVBL1 expression was not apparent and the highest levels of expression were in the developing mouse heart. RUVBL1 was widespread throughout the atrial chambers and primitive ventricle, and in the developing vasculature (Fig. 5B).

**Figure 6.**
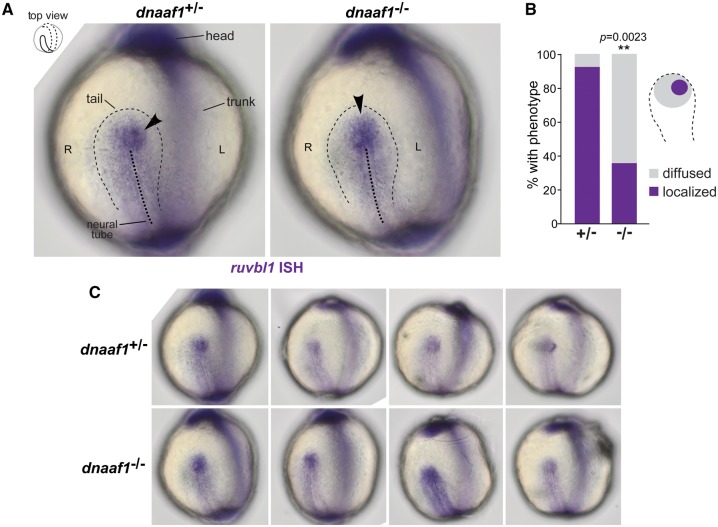
DNAAF1-dependent left-sided asymmetric expression of RUVBL1 at the zebrafish Kupffer’s vesicle: (**A**) *in situ* hybridization (ISH) of *Ruvbl1* expression at Kupffer’s vesicle (thin dashed black line) in wild-type heterozygous *dnaaf1^+/−^* and mutant homozygous *dnaaf1^−/−^* zebrafish embryos, at the 5 to 9 somite stages, visualized from dorsal views as indicated in the top left inset. Localized expression is indicated by arrowheads, and the mid-line of the notochord is shown by the thick dashed black line. Left (L) and right (R) sides are indicated. (**B**) Bar graph shows a significantly higher proportion of wild-type zebrafish (+/−) have left-sided localisation of *ruvbl1* when compared to diffused central localization in mutant (−/−) embryos (*P *=* *0.0023, unpaired Student’s t-test). (**C**) Additional examples of *ruvbl1* expression at Kupffer’s vesicle in unaffected *dnaaf1^+/^*^−^ heterozygous and *dnaaf1^−/−^* affected mutant zebrafish embryos.

To substantiate an *in vivo* functional association between DNAAF1 and RUVBL1, we used ISH to visualize *ruvbl1* expression in wild-type and *dnaaf1*^*−/−*^ mutant zebrafish embryos at the 5 to 9 somite stages. Preliminary experiments indicated that *ruvbl2* was ubiquitously expressed (data not shown). However, *rubvl1* expression was asymmetric in wild-type zebrafish embryos, with significantly higher expression on the left side of the Kupffer’s vesicle (Fig. 6A–C), consistent with the asymmetric distribution in the mouse embryonic node. In *dnaaf1*^*−/−*^ embryos, this asymmetrical distribution was abolished, and embryos showed a central expression of *ruvbl1*.

## Discussion

The link between PCD and CHD has long been recognized. *Situs inversus* occurs in around 50% of patients with PCD, usually with a total mirror-image arrangement of the abdominal organs including the heart, an arrangement that is not usually pathological. However, CHD is more common in PCD than in the general population and one study demonstrated heterotaxy in 6.3% of >300 PCD patients, including 8 patients with complex cardiovascular lesions (14). Mouse models of PCD show even higher rates of complex CHD (27,28), suggesting that in humans *in utero* loss of embryos with severe CHD could account for this difference. This appears to hold true in *inversus viscerum* (*Dnah11^iv^*) mutant mice, reported as a model of PCD (29), where a 40% incidence of CHD is seen prenatally but only 5% is seen in viable term animals (30). Nakhleh *et al.* (2012) demonstrated that the converse was also true, since in a cohort of patients with heterotaxy, 42% were found to have ciliary dysfunction, similar to that seen in PCD (16). These patients had low NO levels, or levels bordering the cut-off values indicative for PCD. Genomic sequencing in these patients demonstrated an increased burden of non-coding variants in PCD genes, but none was found to have biallelic pathogenic mutations in a PCD gene. There was a high level of single heterozygous PCD gene mutations, as well as mutations in two different PCD genes in the same patient. It was suggested that the additive effects of this mutational load may therefore play a role in the aetiology of heterotaxy in these patients (16). A recent unbiased forward genetic screen across the mouse genome for recessive mutations causing congenital heart defects, revealed a high mutational burden in cilia-related genes, including mutations in PCD genes and other cilia motility factors (28).

In this study, we describe four families with mutations in the known PCD gene, *DNAAF1*. Three have affected children with a typical PCD phenotype arising from mutations in *DNAAF1* predicted to truncate the protein, causing a total loss of DNAAF1 protein. As a phenotypic series, there are insufficient numbers to calculate statistical significance but there appears to be a high incidence of CHD in these families, with 5 of 8 patients having complex CHD. Cardiac defects have not been noted in the seven previously reported PCD families with *DNAAF1* mutations (17,18), but our families appear to have a higher rate than would be expected in an unselected PCD cohort, for which the incidence of CHD is currently estimated at 3.5–6% of cases (14). Moreover, we also describe a family with a novel *DNAAF1* missense variant, p.Leu191Phe, that only manifests a cardiac phenotype. This variant fulfils the major criteria for a pathogenic mutation, and its pathogenicity was confirmed by the absence of genetic complementation in *dnaaf1*^*−/−*^ mutant zebrafish embryos (Fig. 2). Both affected siblings in Family A (Fig. 1A) had complex phenotypes of isolated CHD without any clinical manifestations of typical PCD: neither of the two affected siblings have suffered the respiratory symptoms that usually indicate PCD. Ciliary beat frequency and EM were normal and refuted a clinical diagnosis of PCD in this family. These observations suggest that CHD and PCD form a phenotypic continuum rather than comprising distinct clinical entities. The majority of biallelic *DNAAF1* mutations previously associated with PCD were reported to be loss-of-function alleles (17,18), and we suggest that the homozygous p.Leu191Phe mutation may be hypomorphic since it causes isolated CHD. However, further work is required to understand whether this missense mutation has significantly different cellular effects compared to loss-of-function alleles causing CHD and PCD. Our *in vitro* data (Fig. 3B) indicates that the p.Phe191 mutation causes reduced DNAAF1 protein levels, presumably due to instability or increased degradation of the mutant protein. We expect that further genetic screening will identify additional patients with isolated complex CHD that carry mutations in PCD genes, even if respiratory ciliary function is apparently normal. Identification of these genetic aetiologies may thereby refine prognosis, disease stratification and categorization during the medical and surgical management of these patients (31). This is of potential clinical importance because complex CHD carries a high burden of post-surgical morbidity and mortality (31). It is also important to note that DNAAF1 genotype-phenotype correlations are likely to be unusually broad because heterozygous germline mutations in *DNAAF1* (for example, p.Gln307Glu) are associated with an increased risk of testicular germ cell tumours in humans (23,32,33), and rare heterozygous missense mutations are also associated with neural tube defects (33).

Our observation of a phenotypic continuum between CHD and PCD raises questions about the pathomechanism of the p.Leu191Phe missense mutation. This mutation appears to affect nodal ciliary function, manifesting as heterotaxy and CHD, whereas respiratory ciliary function is normal. Two possibilities could explain this observation. Firstly, it may be that subtle defects in motile ciliary function are present but were not identified by standard clinical investigations. These subtle defects may be sub-clinical in their effect on respiratory cilia and mucociliary clearance, but could still disrupt nodal ciliary function because of the exquisitely sensitive nature of coordinated ciliary beat at the node during embryonic development. Secondly, the missense mutation could affect DNAAF1 functions that are distinct from its known role in the assembly of dynein arms. To test the latter possibility, we sought to identify proteins that interact with DNAAF1 in order to infer new putative functions. Yeast two-hybrid and co-immunoprecipitation experiments identified and confirmed that DNAAF1 interacts with the intraflagellar transport protein IFT88 (Fig. 3A–C). As a component of the IFT-B complex, IFT88 is essential for the trafficking of protein cargoes along axonemal microtubules (34). This suggests that DNAAF1 could mediate the delivery of dynein arm components directly to the intraflagellar transport complexes. This is supported by immunofluorescence studies that demonstrate the co-localization between DNAAF1 and ciliary IFT88 (Fig. 4A and B, Supplementary Material, Fig. S3A and B). TAP and co-immunoprecipitation experiments also confirmed interactions of DNAAF1 with both RUVBL1 (Table 1, Fig. 3D) and RUVBL2 (Table 1, Supplementary Material, Fig. S1).

**Figure 7.**
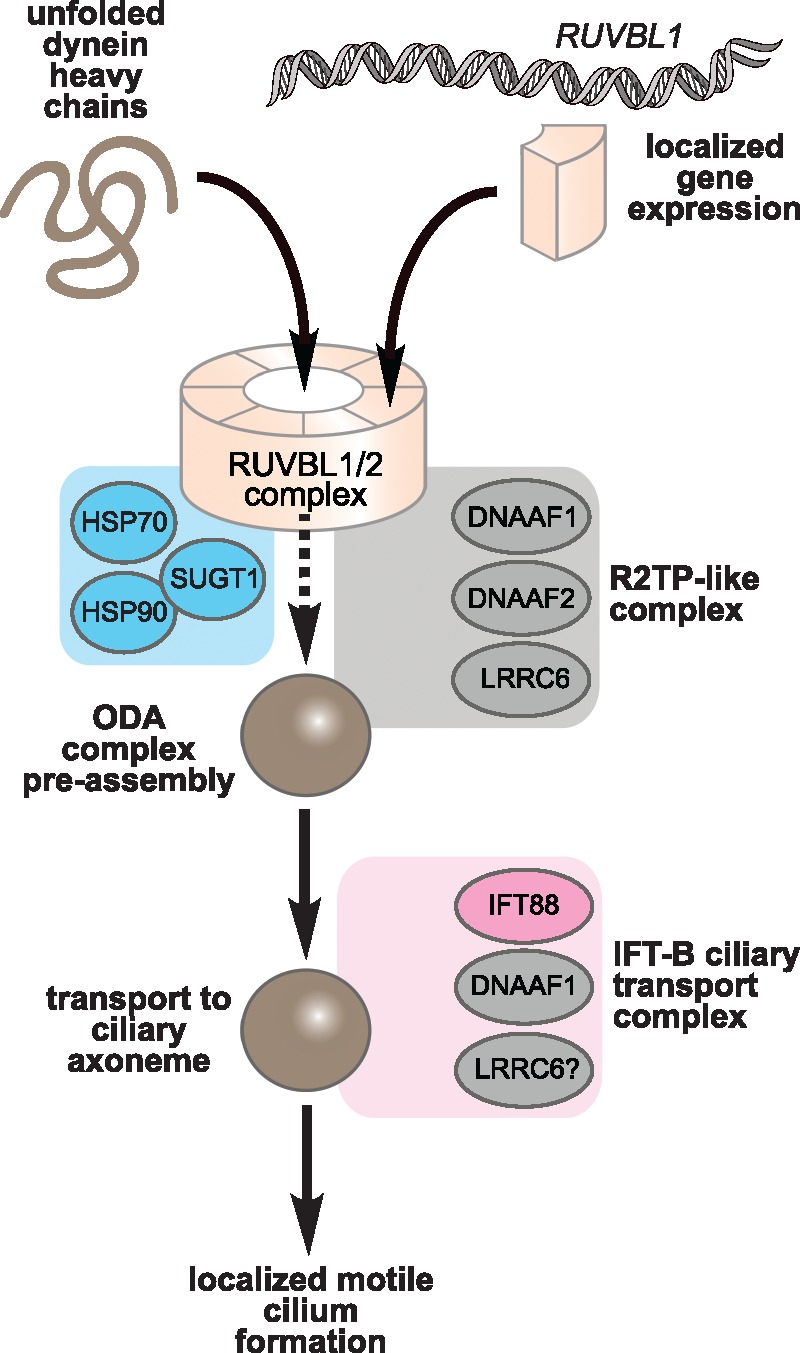
Proposed model of DNAAF1 function in an RT2P-like complex and the IFT-B ciliary transport complex: Unfolded dynein arm components (brown) are folded via the action of DNAAF1 within an ODA pre-assembly module consisting of the AAA+ ATPases RUVBL1 and RUVBL2 (tan), RT2P-like complex proteins (grey) such as DNAAF1, DNAAF2/KTU, and other chaperone proteins (blue) such as SUGT1 and HSP90. In addition to dynein arm assembly through an RT2P-like complex, DNAAF1 also associates with the IFT-B complex (pink; represented by IFT88) allowing dynein arm assembly to be coupled to ciliary transport. RUVBL1 may also have a separate, direct gene regulatory role on asymmetrically expressed genes such as Nodal.

RUVBL1 and RUVBL2 are DNA-dependent AAA+ ATPases involved in numerous cellular processes, and are essential components of protein complexes that mediate chromatin remodelling, transcription factor function, and snoRNP assembly and maturation (35). RUVBL1/RUVBL2 form hetero-hexamer and dodecameric ring-like structures that are the core of many complexes, including the RT2P complex, which is important in the biogenesis of snoRNPs and telomerase. RUVBL1 and RUVBL2 form this complex with Pih1 (Protein interacting with Hsp90) and Tah1 (TPR-containing protein associated with Hsp90), two Hsp90 interactors (36). Interestingly, our TAP experiments demonstrated that HSP90 and SUGT1 both interact with DNAAF1. SUGT1 is a TPR repeat-containing protein and a known co-chaperone of HSP90 that interacts with many LRR domain-containing proteins (37). That DNAAF1 binds RUVBL1/RUVBL2 might indicate a putative model of interaction where an RT2P-like complex is formed from RUVBL1/RUVBL2, a TPR-repeat containing protein (for example SUGT1) and a PIH1-domain containing protein. In this model, appropriate PIH1 domain-containing proteins could include DNAAF2/KTU and MOT48, both linked to cytoplasmic pre-assembly of dyneins (38–40). Furthermore, DNAAF1 appears to associate with IFT-B complex (represented by IFT88) at the ciliary base, suggesting that it is connected directly to IFT of the ODA into cilia and providing an explanation for the previous observation of small amounts of DNAAF1 in ciliary axonemes (18). We hypothesise that DNAAF1 mediates both dynein arm assembly through an RT2P-like complex, and is then further required to couple this process to the IFT-B machinery (Fig. 7). In support of this hypothesis, we observed that loss of RUVBL1 perturbed the co-localization of DNAAF1 with IFT88 in the cilium (Fig. 4B).

Both RUVBL1 and RUVBL2 have previously been implicated in cardiac development in zebrafish (41), but our model implies that RUVBL1/RUVBL2, in addition to other roles, also have a more specific ciliary assembly function. In support of this model, RUVBL1/RUVBL2 interact with other ciliary leucine-rich repeat proteins in addition to DNAAF1 (which is also known as LRRC50). LRRC6/Seahorse is another dynein arm assembly factor (42), and a recent study identified an interaction between RUVBL2/Reptin and LRRC6/Seahorse (43). Consistent with our model, zebrafish embryos with a loss-of-function *ruvbl2* mutation had a typical ciliopathy phenotype (43), including ventral body curvature and kidney cysts, that are features also observed in *ruvbl1* mutants (44). Zebrafish *ruvbl2* mutants had an absence of dynein arms in motile cilia, suggesting that RUVBL2 mediates dynein arm stability and assembly or maintenance (43). However, RUVBL1 evidently has other direct or indirect roles in these processes because we unexpectedly observed asymmetric left-handed distribution of RUVBL1 both at the embryonic node in mice and the Kupffer’s vesicle in zebrafish embryos (Figs 5A and B, 6A). The asymmetric distribution was dependent on DNAAF1 (Fig. 6A–C), indicating that the DNAAF1-RUVBL1 association has a possible functional role in symmetry breaking.

To explain these observations, one possibility is that dynein arm assembly in nodal cilia is initially specified on the left of the node, at least during the early formation of the embryonic node. In this model, the earlier assembly or maturation of nodal cilia on the left would contribute to establishing leftward nodal fluid flow. As a consequence, earlier dynein arm assembly on the left of the node could then ensure that immotile cilia on the perinodal crown cells in this region respond to nodal flow before those on the right of the node. Furthermore, the modulation of cilia motility between the two sides of the node could also act to amplify any early asymmetry imposed by the initial weak levels of leftward nodal flow (45). This hypothesis is supported by the existence of interlinked feedback loops for regulating the expression of Cerl2 and Wnt3, two key mediators of the flow signalling cascade, that could amplify a small initial signal (46). These possibilities do not exclude a direct gene regulatory role for RUVBL1: it could, for example, also act as a transcriptional activator of asymmetrically expressed genes such as Nodal, which is preferentially expressed on the left side of the node. Our intriguing observation of asymmetric distribution of RUVBL1 at the node requires further investigation in animal models in order to understand the unexpected role of RUVBL1 (and possibly RUVBL2) in left-right axis determination.

In summary, we have shown that biallelic mutations in a known PCD gene, *DNAAF1*, are associated with isolated CHD, in the absence of any clinical evidence for PCD. This finding confirms a shared aetiology between PCD and CHD that has been suggested but not proven in previous studies. This phenotypic continuum provides new insights into the pathogenesis of isolated CHD, suggesting the existence of a putative dynein arm assembly complex that contains DNAAF1, IFT88 and RUVBL1. It also highlights a possible functional association between DNAAF1 and RUVBL1 during the establishment of embryonic laterality that merits further investigation.

## Materials and Methods

### GenBank accession numbers

DNAAF1: RefSeq NM_178452.5 and NP_848547.4

### Patients and families

Families with individuals affected with Congenital Heart Disease were recruited and underwent detailed clinical assessment. Additional families with mutations in *DNAAF1* were ascertained through the national PCD diagnostic service, either at University College London or the Leeds Genetics Laboratory. These families had a clinical diagnosis of PCD confirmed by standard clinical diagnostic testing and symptoms of chronic respiratory disease. Ethical approval for molecular genetics research studies was given by Yorkshire Research Ethics Committee (reference no. 11/H1310/1). Full, written, informed consent was provided by all participating individuals and families. Genomic DNA was extracted from peripheral blood leukocytes by standard salt extraction.

### Clinical testing for PCD

Nasal nitric oxide (nNO) levels were measured by a chemiluminescence analyser and are reported in nl/min. Two NO measurements were taken per nostril during breath holding, with the mean of these being taken as the nNO level. Ambient NO level was recorded before each test. Respiratory epithelial cells were then obtained by nasal brush biopsy and suspended in Medium 199. High speed video and transmission electron microscopy were used to analyse ciliary motility and structure, as previously reported (47). At least 100 cilia cross-sections analysed in TEM where available.

### Homozygosity mapping

Affymetrix 6.0 SNP microarray analysis v.6.0 (Affymetrix) was performed for both affected individuals in Family A. Data analysis was carried out using SnpViewer v0.9.2 (https://sourceforge.net/projects/snpviewer/) to identify shared regions of homozygosity. This created a visual output plus numerical chromosome coordinates of regions of interest.

### Whole exome sequencing

WES was performed by three separate centres (see Supplementary Material). In Family A, WES was undertaken with Agilent SureSelect V5 Human All Exon libraries (Agilent Technologies, Santa Clara, CA, USA) and 100 bp paired-end sequencing was performed on the Illumina HiSeq2000 platform (Illumina, San Diego, USA) or the Illumina Miseq. FASTQ files were aligned to GRCh37 by Novoalign (Novocraft Technologies). .sam and .bam files were processed using SAMtools (48), Picard (Broad Institute) and the Genome Analysis Toolkit (GATK, Broad Institute, Cambridge, MA) (49). GATK’s Haplotype Caller was used to call variants. Recalibration was performed as per GATK’s guidelines (50). Ensembl’s variant effect predictor was used to determine the functional consequences of variants (51). In-house generated perl scripts (see Web Resources; date last accessed October 2017) were used to remove variants with a Minor Allele Frequency (MAF) of 1% or higher present in dbSNP 138 and previous, NHLBI Exome Sequencing Project (ESP) Exome Variant Server, the Exome Aggregation Consortium (ExAC) and gnomAD databases, and over 3000 ethnically-matched locally-sourced control samples. A similar pipeline was followed for variant calling in Family B (24), C & D (25). Variants were retained that were predicted to have a functional effect on the protein, including missense, splice site variants and indels. Variants were then retained which followed the predicted inheritance pattern in the family. Variants were retained if predicted “pathogenic” by any one of Polyphen2 (52), SIFT (53) or Condel (54). Finally variants were ranked by CADD score (55) and those with CADD score >15 were retained. Sanger sequencing was performed using standard methodologies. Primer pair sequences are available on request.

### Whole genome sequencing and breakpoint analysis

Approximately 1 µg of genomic DNA was sheared using a CovarisS2 (Covaris, Inc., Woburn, MA, USA). The fragmented DNA was used to create an Illumina compatible whole genome sequencing (WGS) library with NEBNext Ultra reagents, following manufacturer’s protocols throughout (New England Biolabs, Ipswich, MA, USA). The library insert size selection was 300–400bp and the final enrichment PCR consisted of 6 rounds of PCR. Library quality was checked using an Agilent Bioanalyser (Agilent Technologies, Santa Clara, CA, USA) before sequencing was performed on a single lane of an Illumina HiSeq3000 (Illumina, San Diego, CA, USA), which generated paired-end 151bp reads. Raw sequence data were converted to FASTQ.gz format using bcl2fastq v.2.16.0.10, and the quality of these data was assessed using FastQC v.0.11.5 (see Web Resources; date last accessed October 2017). Adaptor sequences and low-quality bases (Q score ≤10) were trimmed from sequence reads using Cutadapt v.1.9.1 (see Web Resources) before being aligned to an indexed human reference genome (hg19) using BWA MEM v.0.7.13 (56). Reads were sorted by chromosome coordinate and PCR duplicates were marked using Picard v.2.1.1 (see Web Resources; date last accessed October 2017). Aligned sequence reads were visualized using the Integrative Genome Viewer v.2.3.80 (57). Alignments mapping to the *DNAAF1* locus were extracted for interrogation using samtools v.0.1.18 (48) to identify putative breakpoint spanning reads. BLAT was used to determine the genomic coordinates of both the 5’ and 3’ sequences (58).

A PCR amplicon was optimized to amplify across the identified breakpoint using primers 5’-dTGTAAAACGACGGCCAGTTGTTCTGGAGGGACGATGAG-3’ (common forward) and dCAGGAAACAGCTATGACCTTGTTGTGGCAGCTGTGAAA (deletion reverse), which generated a 377-bp PCR product. A second reverse primer 5’-dCAGGAAACAGCTATGACCAGCCATCAAGCCTATTTCCAA-3’ (reverse normal) was designed to work in combination with the common forward primer to amplify a larger 551-bp PCR product specific to the normal allele. All primers contained universal tags (underlined) to enable Sanger sequencing using our diagnostic laboratory workflow. Each PCR consisted of 0.5 µl DNA (1 µg/µl), 11µl MegaMix (Microzone Ltd., Haywards Heath, UK), 1 µl of 10 µM common forward primer and 1 µl of 10 µM reverse deletion or reverse normal primer. For each PCR thermocycling conditions comprised a pre-heat step at 94 °C for 5 min followed by 30 cycles of 94 °C for 30 s, 55 °C for 1 min, and 72 °C for 45 s. A final extension step was carried out at 72 °C for 5 min.

### CNV analysis

The ExomeDepth package (see Web Resources) was used to assess CNVs in exome data from Family A. This program compares read depth between the test sample and 5–10 control samples. This was annotated to indicate common CNVs from control populations (59). CNV calls were then ranked by the Bayes Factor (the log10 likelihood ratio of data for the CNV call divided by the normal copy number). CNV calls were manually inspected to assess read ratio and the genes affected by dosage changes. The genes were cross-referenced with current literature, cilia-related gene databases and exome call data for each sample.

### Gateway cloning

The c.571C > T mutation was inserted into a DNAAF1 Gateway entry clone to form a mutant construct by site-directed mutagenesis (SDM) using the Q5 site-directed mutagenesis kit and protocol (New England Biolabs). A SNP of unknown function, c.1634T > G, was removed by SDM. Following this, Gateway LR Clonase reaction (Thermo Fisher Scientific) was performed to subclone mutant and wild-type cDNA into entry vectors pCS2+ and GW331 for SF-TAP. Bacterial transformation and mini/maxi preps (Qiagen) were performed for construct growth and DNA extraction. All cDNA clone sequences were confirmed by Sanger sequencing. Primer sequences available on request.

### Zebrafish mRNA transcription and microinjection

Microinjection and analysis of *dnaaf1*^*−/−*^ zebrafish was performed as previously described (21). pCS2+ constructs were linearized by digestion with *Not*I. mRNA was created by SP6 mMessage mMachine kit (Ambion). Microinjection was performed according to previously published methods (21,60) into the yolks of 1–4 cell stage embryos. For *in situ* hybridization, zebrafish embryos were fixed at the 5–9 somite stage.

### Zebrafish whole mount *in situ* hybridisation

*ruvbl1* probe DNA template was amplified from zebrafish cDNA using forward GAAGGAGAGAGTGGAGGTCG and reverse CTCTTCGCTGATGTTGAGGC primers and cloned into pGEMT vector (Promega) according to standard protocol. Antisense RNA probe was generated using T7 RNA polymerase and purified by ammonium acetate precipitation. Zebrafish embryos were collected from *dnaaf1^+/−^* heterozygote- heterozygote intercross matings and developed until the 5-somite stage, after which they were fixed overnight with 4% PFA at 4 °C. Embryos were washed with PBST (PBS plus 0, 1% Tween20) and gradually (in steps of 25%) brought to 100% methanol and stored at − 20 °C (at least 24 h). Upon start of the *in situ* hybridization protocol, embryos were rehydrated to 100% PBST (in steps of 25%) and treated with proteinase K (10 µg/ml; Promega) for ∼1 min and re-fixed in 4% PFA for 20 min. Embryos were washed in PBST (3 times, 5 min) and prehybridized in Hyb+ (de-ionized formamide 55–60x, SSC 5x, Heparin 50 µg/ml, tRNA 500 µg/ml, Tween20 0, 1%, Citric Acid to pH6) at 68 °C for at least 2 h. Prehybridization mix was replaced with preheated Hyb+ mix containing ∼200–300ng of RNA probe and incubated over night at 68 °C. The next day, embryos were washed with Hyb- (Hyb+ minus Heparin and tRNA) to 100 2X SSC (in steps of 25%) for 15 min at 68 °C and subsequently with 0.2X SSC to 100% PBST (in steps of 25%). Next, embryos were treated with blocking solution (10% blocking buffer, Roche) for at least 2 h and subsequently incubated with anti-DIG antiserum (1: 2000 anti-DIG Fab, Roche) at 4 °C overnight. Unbound antibodies were washed away with six PBST washes (each 15 min). Finally, PBST was replaced with staining solution (0.1M TrisHCl pH9.5, 0.05M MgCl2, 0.1M NaCl, 0.1% Tween20). Staining was performed with NBT/BCIP (stock solution, Sigma) and embryos were monitored every 15–30 min. When staining was optimal, embryos were washed with PBST and brought to 100% methanol (in steps of 20%) and stored at − 20 °C for at least 24 h. Embryos were imaged in 100% glycerol using IX53 inverted microscope (Olympus). Genotyping was performed as described below.

### Zebrafish DNA isolation and genotyping

For isolation of DNA, early stage zebrafish embryos were dechorionated and lysed in 100 µl DNA extraction buffer (10 mM Tris pH8, 2 mM EDTA, 0, 2% Triton X-100, 200 µg/ml proteinase K) for 60 min at 65 °C. Proteinase K was inactivated by boiling at 95 °C for 15 min. 1 µl of DNA template was used in PCR reaction for *dnaaf1* genotyping with forward CCTGTATCCTCTTCAGAATTACCAA and reverse GGGCCAAAGTTTGACAATGA primers. Amplified PCR products were sequenced using EZ-Seq (Macrogen) and analysed using GeneStudio software.

### Tandem affinity purification

HEK293 cells at 70% confluence were transfected with mutant (p.Phe191) or wild-type (p.Leu191) DNAAF1 SF-TAP constructs using Lipofectamine 2000 (Thermo Fisher Scientific), following the manufacturer’s guidelines. After 4 h media was changed for DMEM-F12 + 10% foetal calf serum (FCS). At 48 h, cells were collected in 1 × PBS and lysed in lysis buffer (50 mM Tris-Cl pH8.0, 150 mM NaCl, 1%[v/v] NP40, 0.5 mM EDTA) supplemented with protease inhibitors (Thermo Fisher). Lysates were centrifuged at 200 × *g* for 5min at 4 °C. Approximately 2 mg of lysate was incubated with 100 µl of washed streptactin “Superflow” beads for 1.5 h at 4 °C with end-to-end mixing. The beads were pelleted at 5000 × *g* and the supernatant discarded. Streptactin beads were transferred to a microspin column and excess fluid was removed by gravity. 500 µl ice-cold wash buffer (1xTBS, 1xprotease-phosphatase inhibitors, 0.1%[v/v] NP40) was added, and the column spun down for 10s at 1000 × g. This wash was repeated a further two times. Bound proteins were eluted with 200 µl ice-cold D-desthiobiotin elution buffer with a 15 min incubation on ice: 100 mM Tris HCl pH8, 150 mM NaCl, 1 mM EDTA, 2.5 mM to 10 mM D-desthiobiotin. This reaction was transferred to new microspin columns and centrifuged at 1000 × *g* for 10 s to elute proteins. The eluate was incubated with 50 µl of washed Anti-FLAG M2 affinity gel beads (Sigma Aldrich) for 1.5 h with end-over-end mixing. The beads were pelleted at 1000 × *g*, washed with 500 µl of ice-cold wash buffer three times, and washed once with 500 µl ice-cold 1xTBS/PBS. Bound proteins were eluted with 200 µl of ice-cold FLAG peptide elution buffer [200 µg/ml FLAG peptide (Sigma Aldrich) in 1xTBS/PBS] for 15 min on ice. Beads were pelleted at 1000 × *g* and the eluate transferred to a fresh tube. Precipitated proteins were sent for mass spectrometry.

### Yeast two-hybrid interactions

A GAL4-based yeast two-hybrid system (HybriZAP, Stratagene, USE) was used to identify proteins that interact with DNAAF1. The DNA-binding domain fused to the human DNAAF1 (pBD-DNAAF1) was used as bait for screening a human oligo-d(T) primed retina cDNA library containing 1.9 × 10^6^ primary clones fused to the activation domain (pAD). Clones were plated on amino acid dropout plates lacking Trp, Leu and His, containing 1 mM 3-aminotriazol, and selected for growth. Clones were then patched on medium additionally lacking adenine and selected for growth and α-galactosidase activity by the activation of the MEL1 reporter gene. The latter was done using 20 mg/ml of the chromogenic substrate 5-bromo-4-chloro-3-indolyl α-D-galactopyranoside (X-α-Gal) in the dropout plates and selecting the positive clones that developed a blue-green colour. Further selection of positive clones was based on β-galactosidase activity by the activation of the LacZ reporter gene, which was detected by a filter-lift assay.

### Co-immunoprecipitation

HEK293 cells were transfected with SF-TAP-DNAAF1 mutant (p.Phe191) or wild-type, and co-transfected with or without an eYFP-IFT88 construct, using Lipofectamine 2000 (Thermo Fisher Scientific) according to the manufacturer’s protocols. After 4 h the media was changed to DMEM-F12 + 10% FCS. At 48hr, cells were collected in 1 × PBS and lysed in lysis buffer [50 mM Tris-Cl pH 8.0, 150 mM NaCl, 1%[v/v] IGEPAL-630 (Sigma-Aldrich), 0.5 mM EDTA] supplemented with protease inhibitors (Thermo Fisher).

The lysate was centrifuged at 14, 000 × g for 15 min. Protein concentration was determined using a Bradford assay. 1500 µg of protein was pre-cleared with 30 µl of washed protein A-Sepharose beads (Roche) for 30min at 4 °C. Beads were removed by centrifugation at 1000 x g at 4 °C for 1min. 1 µg of the appropriate antibody was incubated with the supernatant for 2 h at 4 °C on an orbital shaker. The incubation buffer consisted of 50 mM Tris-HCl pH 8.0, 150 mM NaCl, 0.5 mM EDTA, 1x protease inhibitors (Thermo Fisher Scientific). Immunocomplexes were captured by the addition of 70 µl protein A-Sepharose beads and incubating overnight at 4 °C. The beads were collected by centrifugation at 1000xg and washed three times with 500ul ice-cold wash buffer (150 mM NaCl, 50 mM Tris-HCl pH 8.0, 0.5 mM EDTA, 0.1%[v/v] NP40, 1x protease inhibitors). 20 µl 2%[w/v] SDS was used to elute proteins and left for 30min at room temperature. The sample was then centrifuged at 14, 000 x g for 2min and the supernatant collected. Lysates were electrophoresed in NuPAGE 4–12% Bis-Tris gels and transferred to PVDF membranes. Membranes were blocked in 5%[w/v] non-fat skimmed milk (“Marvel”) in PBST for 2 h. Membranes were washed four times in PBST (10 min incubations) and incubation in primary antibody was performed for 30min (anti-FLAG antibody only) or overnight at 4 °C (all other antibodies). Membranes were washed four times and incubation with the appropriate HRP-tagged secondary antibody was performed for 1 h at 4 °C. Following a final four 1xPBST washes, membranes were developed using SuperSignal West Femto kit (Thermo Fisher Scientific). Images were acquired using Bio-Rad molecular imager gel documentation system and displayed using Image Lab (v. 4.0) analysis software (Bio-Rad, Hemel Hempstead, UK). Full western blots are shown in Supplementary Material, Figure S5.

The following antibodies were used: Mouse monoclonal anti-Reptin (Sigma –Aldrich SAB4200115, 1: 1000), Mouse monoclonal anti-Pontin (Sigma-Aldrich SAB4200194, 1: 1000), mouse monoclonal anti-FLAG, Clone M2 (Sigma-Aldrich F9291, 1: 1000), rabbit IFT88 polyclonal antibody (Proteintech 13967–1-AP, 1: 200), rabbit polyclonal anti-DNAAF1 (Sigma-Aldrich AV53359, 1: 100), mouse monoclonal “Living Colors” anti-GFP (Clontech 632380, 1: 1000). Rabbit polyclonal control IgG (Santa Cruz sc-2027), goat anti-mouse HRP antibody (Dako, 1: 10000) and goat anti-rabbit HRP antibody (Dako, 1: 10000).

### Immunofluorescence and confocal microscopy

Human immortalized hTERT-RPE1 cells were grown in DMEM-F12 + 10% FCS on coverslips in a 6–well plate until 70–80% confluence. Transfection of SF-TAP-DNAAF1 constructs, with or without eYFP-IFT88, was performed with Lipofectamine2000 (Thermo Fisher Scientific) in OptiMEM according to manufacturer’s protocols. After 4hr, media was changed for DMEMF12 + 0.2% FCS and cells were grown for 48 h. Fixation was either with ice-cold methanol (5 min at −20 °C) or 2%[w/v] *para*-formaldehyde (20min at room temperature, then permeabilised with 0.01%[v/v] Triton X-100 for 5 min at room temperature). Coverslips were blocked in 1% milk solution for at least 5min. Primary antibodies were made up in 1% milk solution and incubated for at least 1 h. Coverslips were washed three times in 1xPBS. Incubation with appropriate secondary antibodies in 1% milk solution was performed for 1 h. Following this, five PBS washes were performed. Coverslips were set to slides in Mowiol (Sigma-Aldrich).

Primary antibodies used were: mouse monoclonal anti-Reptin (Sigma-Aldrich SAB4200115, 1: 200), Mouse monoclonal anti-Pontin (Sigma-Aldrich SAB4200194, 1: 200), mouse monoclonal anti-FLAG, Clone M2 (Sigma-Aldrich F9291, 1: 500), rabbit IFT88 polyclonal antibody (Proteintech 13967–1-AP, 1: 200), rabbit polyclonal anti-DNAAF1 (abcam ab75163, 1: 100), rabbit polyclonal anti-DNAAF1 (Sigma-Aldrich AV53359, 1: 100), guinea-pig anti-RPGRIP1L (1: 500), mouse anti-gamma-tubulin (Sigma-Aldrich, 1: 1000), mouse anti-centrin3 (Merck, 1: 100), goat anti-gamma-tubulin (Santa-Cruz sc-7396, 1: 50), rabbit anti-ARL13B (Proteintech, 1: 500) and mouse anti-acetylated α-tubulin (Sigma-Aldrich, 1: 2000). Appropriate Alexa Fluor conjugated secondary antibodies (Life Technologies) were utilized and DAPI (1: 1000) was used for nuclear staining. Confocal images were obtained using a Nikon A1R confocal microscope, processed by NIS-Elements Confocal 4.5, (Nikon) software. ImageJ was used for post-capture image processing of confocal .nd2 z-stack files. Co-localizations were assessed using the Coloc2 plug-in, using IFT88 staining in the green channel to define regions of interest.

### siRNA knockdown

T47D cells were reverse transfected with 50 nM On-TargetPlus siRNA smartpool oligos (Dharmacon) using Lipofectamine RNAimax (Invitrogen). RNA isolation was performed using an RNA isolation kit as per manufacturer's directions (Qiagen). mRNA probes for *RUVBL1* and *RUVBL2* were from Applied Biosystems. Real time PCR was performed in triplicate using the one-step RT-PCR kit and the 7500 system from Applied Biosystems. siRNA knockdown in RPE1 cells was performed using 50 nM On-TargetPlus “SMARTpool” siRNA oligonucleotides (Dharmacon), concurrently with DNAAF1 construct over-expression using Lipofectamine2000 (Invitrogen), using methods as described above.

### Mouse embryo whole mount *in situ* hybridisation

To produce an *in situ* hybridisation probe, a mouse *Ruvbl1* cDNA clone (MmCD00533571) was sourced from the DNASU Plasmid Repository, Center for Eukaryotic Structural Genomics, University of Wisconsin Madison, USA. This was linearized by restriction digestion with *Apa*I (NEB). An anti-sense RNA probe was synthesised using RNA DIG labelling mix (Roche) and T7 RNA polymerase (NEB). Wild-type C3H/HeH mouse embryos were dissected in PBS at E7.5-E8.5, fixed in 4% PFA for 1 h, washed 3 times in PBS and dehydrated through a methanol series after which they were stored at − 20˚C. Following rehydration, whole-mount in situ hybridisation was performed according to standard protocols. Post-hybridisation, DIG was detected using anti-DIG alkaline phosphatase conjugated antibody (Roche, 1; 500). Expression was visualised using NBT/BCIP (Roche). Flat mounted embryos were photography using a Leica MZ16.5 microscope.

### Mouse embryo collection and immunofluorescence

Wild-type C57BL/6 mouse embryos at embryonic stages E7.0-E8.5 were dissected in PBS + 1% BSA. Fixation was performed in 4% *para*-formaldehyde for 1 h and then samples were washed in three 1xPBS washes. Samples were dehydrated then rehydrated in a series of methanol dilutions for 15min each, in increasing then decreasing concentrations, respectively. Embryos were washed in 1xPBS three times and blocked in 1% FCS for 1 h. Primary antibody was diluted in 1% FCS and incubated with the sample overnight at 4 °C. Three 1xPBS washes preceded incubation with secondary antibody in 1% FCS for 1 h. Samples were imaged using either light-sheet microscopy (on a Zeiss Lightsheet Z1 instrument), or confocal microscopy (Nikon A1R) for which the sample was mounted in Mowiol (Sigma-Aldrich). Mouse monoclonal anti-Pontin (Sigma-Aldrich SAB4200194) was used at 1: 200, with appropriate Alexa Fluor conjugated secondary antibodies (Life Technologies) and DAPI at 1: 1000 for nuclear staining. Secondary antibody negative control staining gave minimal background (data not shown).

### Statistical analyses

Statistical analyses were preformed using InStat (GraphPad Prism). Pairwise comparisons were analysed with Student's two-tailed t-test and one-way analysis of variance (ANOVA) was used for three or more independent data-sets. Contingency tables were analysed with Pearson’s chi-squared test, using two degrees of freedom. Results reported are from at least three independent biological replicates. Error bars on bar graphs indicate s.e.m. The statistical significance of pairwise comparisons shown on bar graphs are indicated by: n.s., not significant, *P < 0.05, **P < 0.01, ***P < 0.001, and ****P < 0.0001. For cell populations, a minimum of 150 cells were counted from 10 separate fields of view. Normal distribution of data was confirmed using the Kolmogorov-Smirnov test.

## Supplementary Material

Supplementary Material is available at *HMG* online.

## Supplementary Material

Supplementary Figures

Supplementary Table S1

Supplementary Table S2
