# Supplementary figures and images for: DNAAF1 links heart laterality with the AAA+ ATPase RUVBL1 and ciliary intraflagellar transport
Source: Hum Mol Genet. 2017 Dec 7;27(3):529–45. doi: 10.1093/hmg/ddx422 (PMC5886296; doi:10.1093/hmg/ddx422)

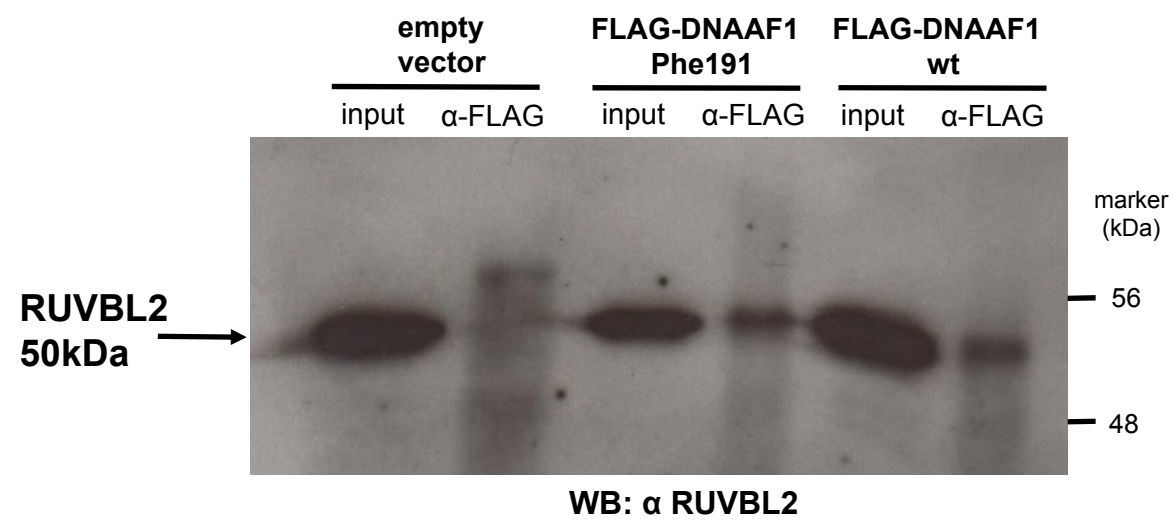

**A**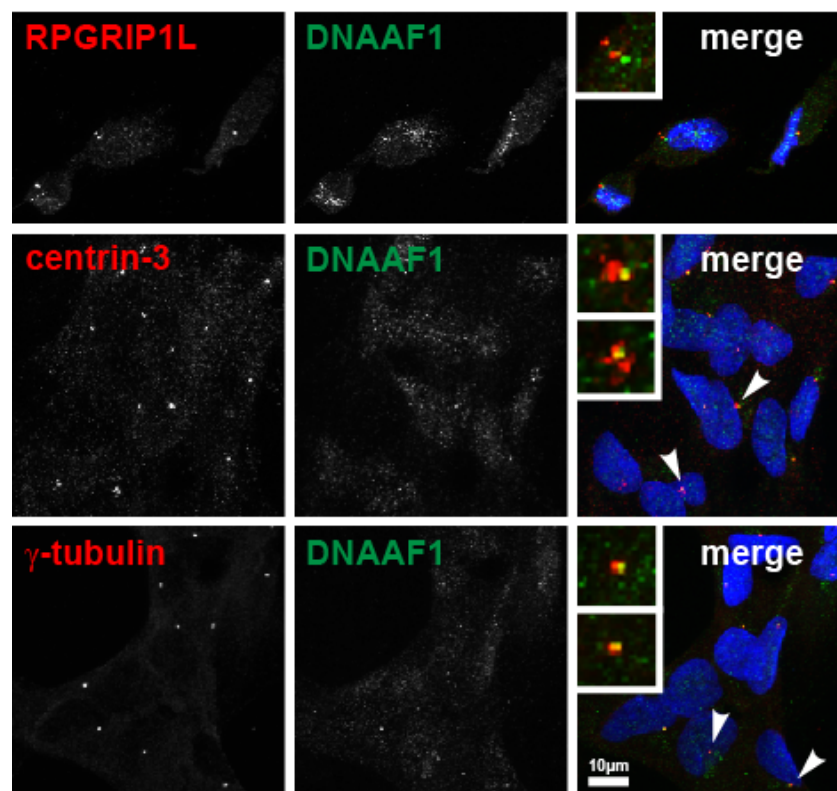**B**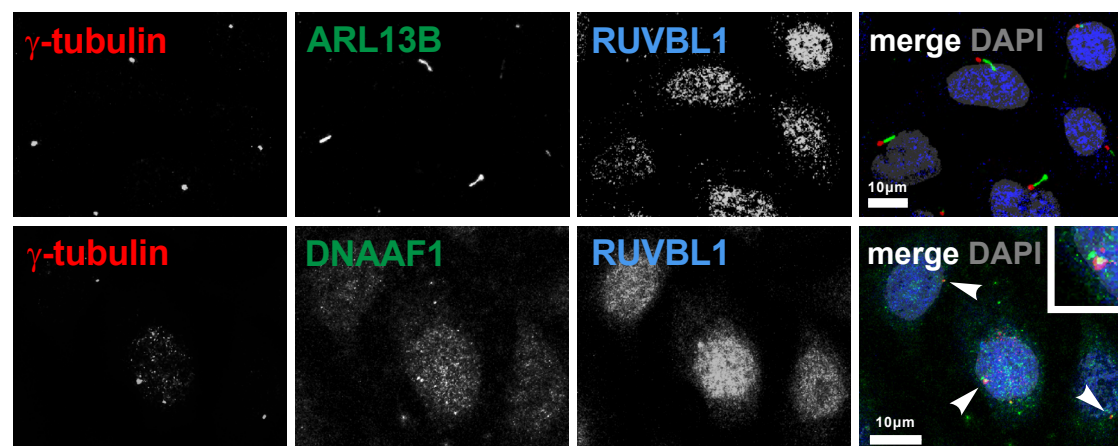

**A**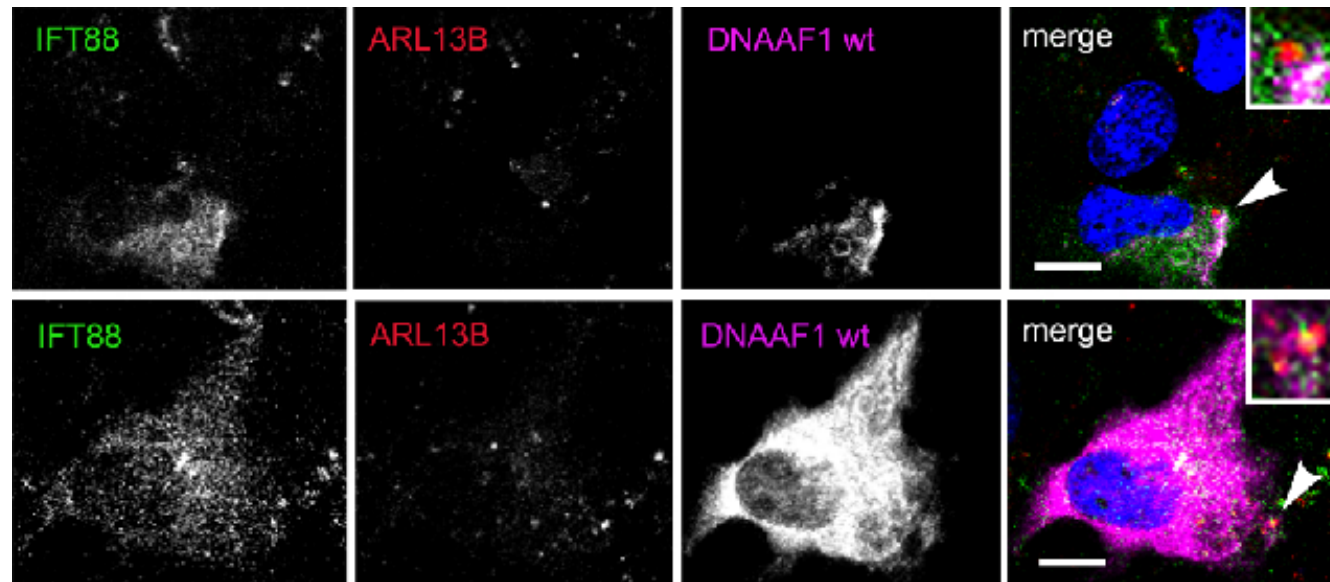**B**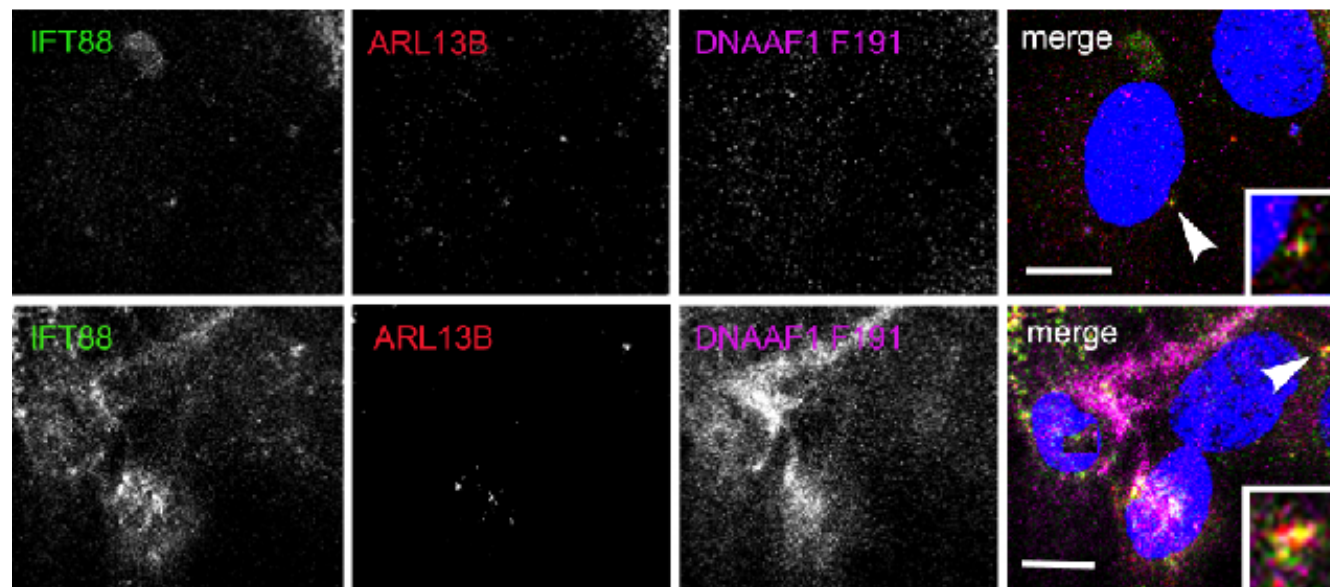

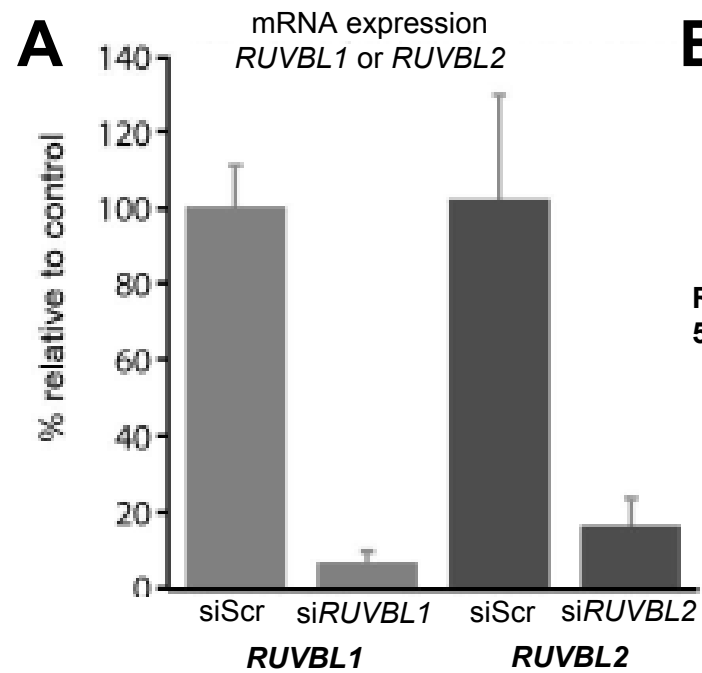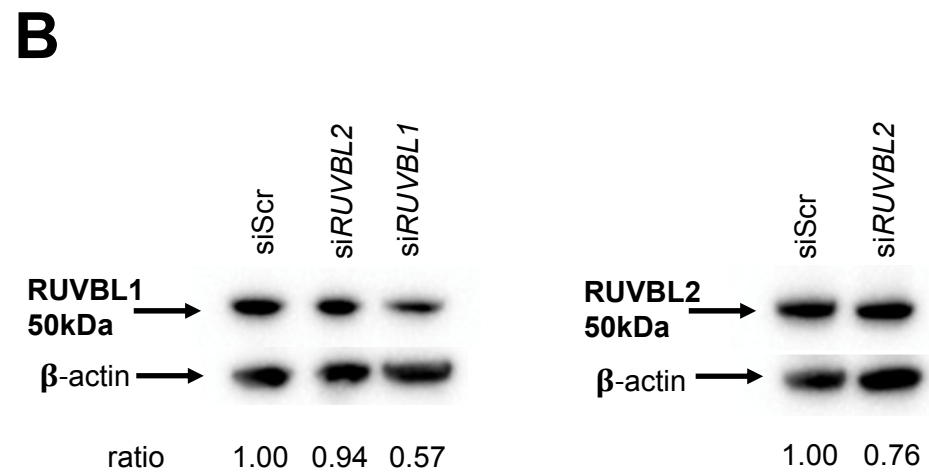

Figure 3B

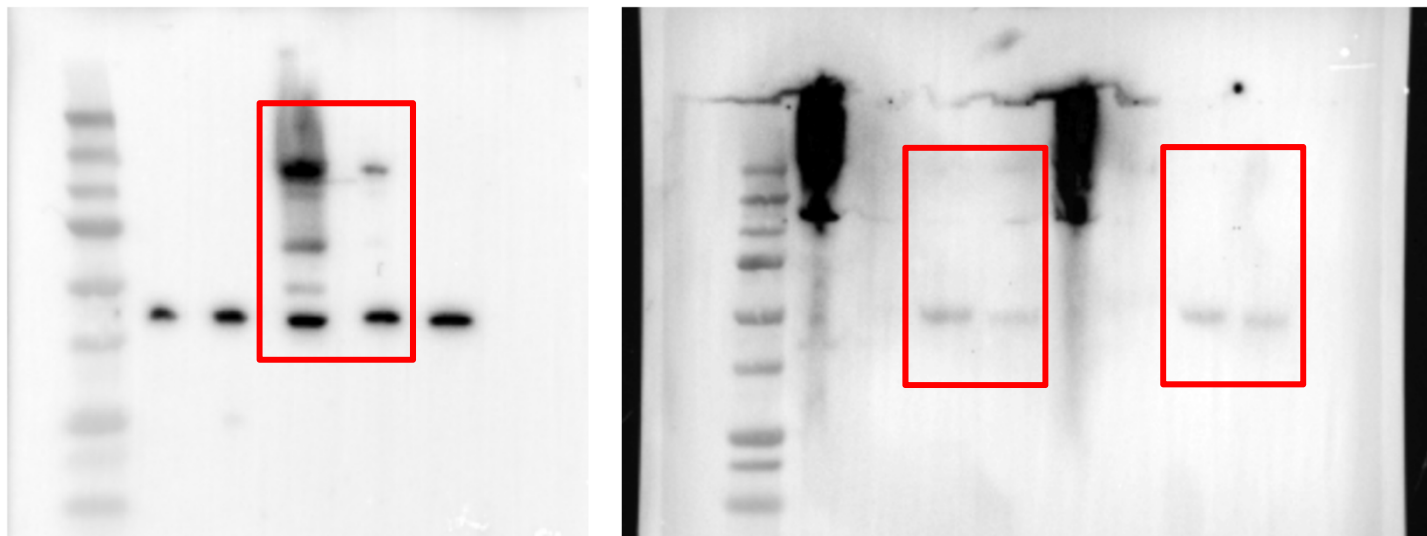

Figure 3C

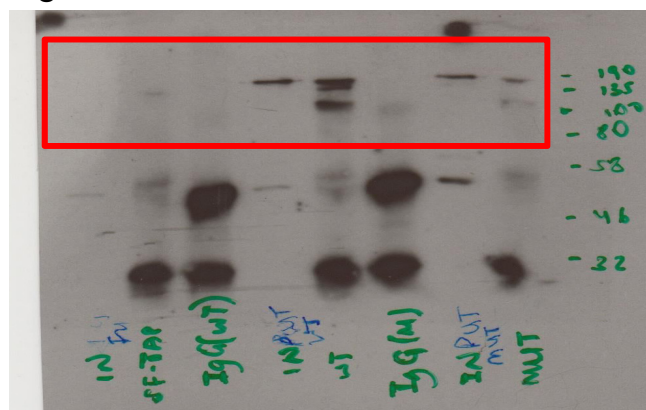

Figure 3D

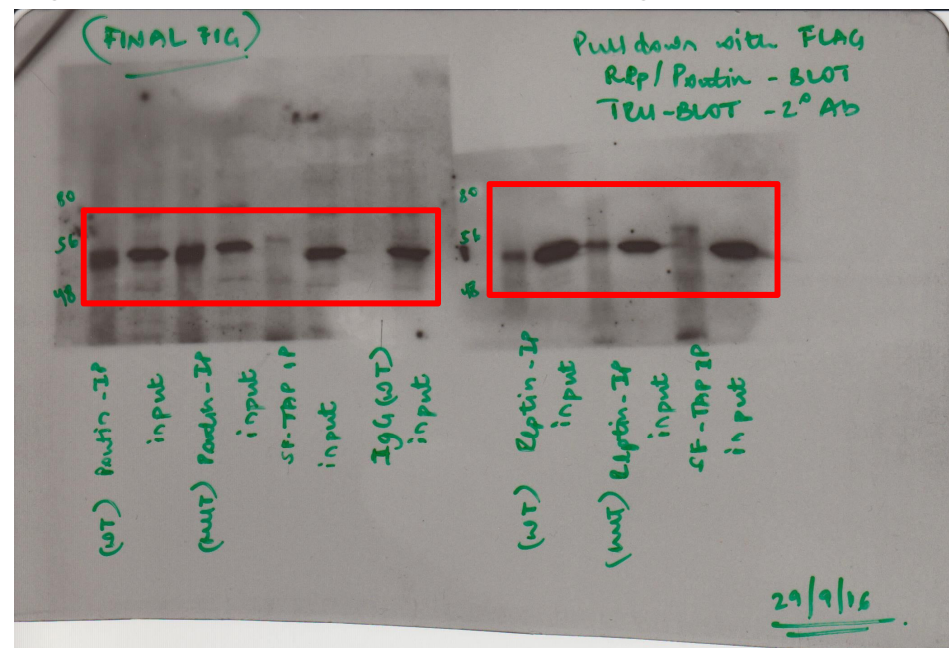

Suppl. Figure 1A

Supplement: Supplementary Figures [file supplementary_figures_ddx422.pdf]
